# Supplementary material for: Effect of Tryptophan Supplementation Levels on the Cecal Microbial Composition, Growth Performance, Immune Function and Antioxidant Capacity in Broilers
Source: Metabolites. 2025 Nov 11;15(11):736. doi: 10.3390/metabo15110736 (PMC12654543; doi:10.3390/metabo15110736)
Supplement: Supplementary file 1 [file metabolites-15-00736-s001.zip › metabolites-3868971-SI.pdf]

Table S1 Relative abundance of species at the phylum level in different tryptophan level treatment groups.

Phylum	Relative Abundance			*P*-value
	Control	Trp_low	Trp_high	
**Firmicutes**	0.541 ± 0.102	0.540 ± 0.207	0.469 ± 0.218	0.783
**Bacteroidetes**	0.276 ± 0.130	0.243 ± 0.121	0.376 ± 0.157	0.317
**Verrucomicrobia**	0.050 ± 0.051	0.158 ± 0.099	0.099 ± 0.147	0.310
**Proteobacteria**	0.123 ± 0.124	0.051 ± 0.049	0.045 ± 0.022	0.243
**Actinobacteria**	0.010 ± 0.004	0.007 ± 0.002	0.010 ± 0.004	0.322
**unclassified**	0.0006 ± 0.0003	0.0016 ± 0.0017	0.0012 ± 0.0011	0.371

Data are presented as mean ± SD (n = 15).

Table S2 The relative abundance of species at the genus level in different tryptophan level treatment groups.

Genus	Relative Abundance			*P*-value
	Control	Trp_low	Trp_high	
Alistipes(Bacteroidetes)	0.264 ± 0.132	0.235 ± 0.120	0.366 ± 0.158	0.323
Lactobacillus(Firmicutes)	0.206 ± 0.053	0.181 ± 0.190	0.184 ± 0.215	0.968
Akkermansia(Verrucomicrobia)	0.050 ± 0.051	0.158 ± 0.099	0.099 ± 0.147	0.310
Escherichia(Proteobacteria)	0.120 ± 0.124	0.049 ± 0.048	0.042 ± 0.023	0.252
Merdimonas(Firmicutes)	0.038 ± 0.017	0.079 ± 0.078	0.035 ± 0.016	0.283
Lachnoclostridium(Firmicutes)	0.040 ± 0.023	0.033 ± 0.015	0.023 ± 0.010	0.291
Erysipelatoclostridium(Firmicutes)	0.029 ± 0.022	0.018 ± 0.008	0.031 ± 0.019	0.468
Sellimonas(Firmicutes)	0.018 ± 0.016	0.015 ± 0.006	0.015 ± 0.003	0.856
Enterococcus(Firmicutes)	0.013 ± 0.014	0.009 ± 0.003	0.023 ± 0.009	0.105
Anaerotignum(Firmicutes)	0.014 ± 0.004	0.017 ± 0.010	0.016 ± 0.007	0.803
Anaerotruncus(Firmicutes)	0.009 ± 0.004	0.020 ± 0.012	0.016 ± 0.013	0.315
Angelakisella(Firmicutes)	0.014 ± 0.005	0.018 ± 0.009	0.010 ± 0.003	0.177
Mordavella(Firmicutes)	0.013 ± 0.006	0.015 ± 0.004	0.011 ± 0.003	0.471
Pseudoflavonifractor(Firmicutes)	0.015 ± 0.006	0.009 ± 0.005	0.009 ± 0.004	0.138
Tyzzerella(Firmicutes)	0.008 ± 0.002	0.010 ± 0.006	0.011 ± 0.005	0.561
Massiliomicrobiota(Firmicutes)	0.009 ± 0.003	0.013 ± 0.019	0.007 ± 0.004	0.747
Clostridioides(Firmicutes)	0.009 ± 0.003	0.009 ± 0.004	0.008 ± 0.003	0.873
Blautia(Firmicutes)	0.009 ± 0.002	0.010 ± 0.007	0.006 ± 0.002	0.365
Ruthenibacterium(Firmicutes)	0.008 ± 0.008	0.009 ± 0.009	0.004 ± 0.005	0.564
Oscillibacter(Firmicutes)	0.015 ± 0.021	0.006 ± 0.004	0.004 ± 0.003	0.353

Data are presented as mean ± SD (n = 15).

Table S3 Effect of dietary tryptophan levels on the abundance of KEGG level 1 pathways.

Item	Abundance			*P*-value
	**Control**	**Trp_low**	**Trp_high**	
Metabolism	2257.45 ± 384.10[b]	3033.31 ± 343.86[a]	3018.54 ± 323.21[a]	0.016
Genetic Information Processing	393.69 ± 49.44	441.44 ± 26.42	418.04 ± 62.83	0.438
Environmental Information Processing	80.77 ± 11.04	89.13 ± 18.37	82.79 ± 15.37	0.700
Cellular Processes	116.26 ± 22.75	131.00 ± 19.30	120.17 ± 23.67	0.612
Organismal Systems	69.28 ± 10.85[b]	86.07 ± 3.52[a]	90.68 ± 12.03[a]	0.027
Human Diseases	172.83 ± 25.30	222.21 ± 35.22	206.44 ± 37.72	0.112

[a,b] Means within the same row sharing the same superscript letter or bearing no superscript indicate no significant difference ($p > 0.05$), and means with different superscript letters indicate a significant difference ($p < 0.05$). Data are presented as mean ± SD (n = 15).

Table S4 Effect of dietary tryptophan levels on the abundance of KEGG level 2 pathways.

Item	Abundance			*P*-value
	Control	Trp_low	Trp_high	
Metabolism				
Amino acid metabolism	295.17 ± 40.22[b]	403.63 ± 24.29[a]	405.13 ± 79.10[a]	0.012
Biosynthesis of other secondary metabolites	145.25 ± 25.54[b]	188.92 ± 8.76[a]	196.01 ± 24.10[a]	0.023
Carbohydrate metabolism	354.28 ± 50.94	399.97 ± 11.73	383.08 ± 56.77	0.430
Chemical structure transformation maps	15.35 ± 8.81[b]	50.79 ± 6.00[ab]	71.75 ± 52.33[a]	0.036
Energy metabolism	140.14 ± 15.66	166.97 ± 12.89	152.05 ± 23.94	0.143
Global and overview maps	177.82 ± 24.91	231.75 ± 19.64	204.15 ± 41.18	0.070
Glycan biosynthesis and metabolism	194.79 ± 93.62[b]	332.53 ± 57.26[ab]	389.46 ± 110.71[a]	0.030
Lipid metabolism	141.49 ± 22.64	186.24 ± 49.02	163.20 ± 29.45	0.242
Metabolism of cofactors and vitamins	301.99 ± 51.94[b]	416.95 ± 14.44[a]	427.40 ± 97.51[a]	0.035
Metabolism of other amino acids	274.47 ± 47.30	294.40 ± 30.73	301.77 ± 82.59	0.779
Metabolism of terpenoids and polyketides	67.52 ± 11.63	90.75 ± 18.87	79.27 ± 15.86	0.124
Nucleotide metabolism	53.69 ± 6.80	57.21 ± 3.68	59.71 ± 11.40	0.556
Xenobiotics biodegradation and metabolism	100.11 ± 18.73	94.18 ± 14.84	102.87 ± 39.27	0.916
Genetic Information Processing				
Folding, sorting and degradation	76.85 ± 8.94	85.13 ± 1.70	82.21 ± 21.29	0.721
Replication and repair	157.84 ± 23.23	185.34 ± 8.61	175.70 ± 39.85	0.430
Transcription	12.35 ± 1.58	11.28 ± 0.87	12.71 ± 2.61	0.617
Translation	146.65 ± 24.13	159.70 ± 15.32	139.92 ± 14.55	0.444
Environmental Information Processing				
Membrane transport	56.23 ± 8.68	52.62 ± 11.65	49.91 ± 11.06	0.647
Signal transduction	24.37 ± 2.94	29.33 ± 2.18	30.04 ± 6.87	0.184
Signaling molecules and interaction	0.17 ± 0.13	0.19 ± 0.17	0.27 ± 0.20	0.638
Cellular Processes				
Cell growth and death	55.28 ± 7.21	66.31 ± 4.00	64.66 ± 14.55	0.283
Cell motility	26.86 ± 11.49	13.72 ± 6.21	9.62 ± 6.81	0.053
Cellular community - eukaryotes	-	-	-	-
Cellular community - prokaryotes	35.25 ± 8.38	31.32 ± 6.84	27.11 ± 4.08	0.200
Transport and catabolism	8.41 ± 3.01	12.92 ± 1.90	12.12 ± 5.30	0.199
Organismal Systems				
Aging	12.19 ± 0.77	15.70 ± 3.85	15.53 ± 4.80	0.255
Circulatory system	-	-	-	-
Digestive system	7.39 ± 2.94	6.48 ± 3.62	7.64 ± 4.62	0.880
Endocrine system	26.13 ± 5.39	26.95 ± 5.29	26.76 ± 2.55	0.965
Environmental adaptation	2.97 ± 0.48	3.08 ± 0.67	3.32 ± 0.63	0.640
Excretory system	7.52 ± 1.90[b]	12.72 ± 2.78[a]	11.81 ± 2.24[a]	0.011
Immune system	3.78 ± 0.47	5.53 ± 1.47	5.14 ± 2.02	0.184
Nervous system	9.30 ± 1.02[b]	13.60 ± 1.77[a]	12.34 ± 3.07[ab]	0.031

Table S4 Effect of dietary tryptophan levels on the abundance of KEGG level 2 pathways. (Continued)

Item	Abundance			*P*-value
	**Control**	**Trp_low**	**Trp_high**	
Sensory system	-	-	-	-
Human Diseases				
Cancers: Overview	20.77 ± 3.19	25.79 ± 6.77	23.46 ± 5.52	0.387
Cancers: Specific types	2.43 ± 0.68	2.43 ± 1.01	2.23 ± 0.87	0.916
Cardiovascular diseases	3.57 ± 0.42	4.68 ± 1.75	4.00 ± 0.86	0.340
Drug resistance: Antimicrobial	78.44 ± 13.90^{b}	101.39 ± 9.79^{a}	105.62 ± 14.25^{a}	0.027
Drug resistance: Antineoplastic	27.10 ± 4.63^{b}	37.61 ± 5.77^{a}	36.93 ± 4.61^{a}	0.022
Endocrine and metabolic diseases	17.31 ± 2.00	21.54 ± 5.13	19.23 ± 3.75	0.278
Immune diseases	4.92 ± 0.85	5.13 ± 1.58	5.07 ± 1.91	0.974
Infectious diseases: Bacterial	14.40 ± 1.59	17.58 ± 5.15	14.74 ± 3.46	0.382
Infectious diseases: Viral	0.95 ± 0.28	1.20 ± 0.21	1.16 ± 0.31	0.368
Neurodegenerative diseases	2.92 ± 0.65	3.34 ± 1.33	3.32 ± 1.28	0.809
Substance dependence	0.01 ± 0.02	0.05 ± 0.10	-	0.354

^{a,b} Means within the same row sharing the same superscript letter or bearing no superscript indicate no significant difference ($p > 0.05$), and means with different superscript letters indicate a significant difference ($p < 0.05$).

'-' Too low to be calculated. Data are presented as mean ± SD (n = 15).

P-values < 0.05 are highlighted.

Table S5 Effect of dietary tryptophan levels on the abundance of KOs in tryptophan metabolic pathway (Metabolism^{1st}→Amino acid metabolism^{2nd}→tryptophan metabolism pathway^{3rd})[1].

KOs	Species origin	Abundance			*P*-value
		Control	Trp_low	Trp_high	
K01667(tnaA)	Akkermansia_muciniphila	13.27 ± 12.86	52.07 ± 33.41	37.19 ± 61.40	0.353
	Escherichia_coli	5.99 ± 5.79	2.65 ± 2.32	3.32 ± 3.16	0.492
	unclassified	62.39 ± 21.70	51.07 ± 26.94	75.48 ± 41.04	0.482
K00128(ALDH)	Gordonibacter_pamelaeae	2.90 ± 1.73	1.85 ± 0.54	4.41 ± 3.16	0.362
K00837(ISS1)	Klebsiella_pneumoniae	0.06 ± 0.13	-	-	-
K20807(nthB)	Klebsiella_pneumoniae	0.07 ± 0.15	-	-	-
K01426(E3.5.1.4)	Enterococcus_faecalis	1.07 ± 1.17	1.46 ± 0.71	4.56 ± 3.98	0.180
	Gordonibacter_pamelaeae	3.41 ± 2.14	3.16 ± 0.22	1.86 ± 0.03	0.522
	unclassified	9.24 ± 5.95	6.16 ± 4.58	7.62 ± 7.16	0.724
K03781(katE)	Akkermansia_muciniphila	12.33 ± 9.67^{b}	53.99 ± 19.49^{ab}	75.60 ± 53.41^{a}	0.041
	Enterococcus_faecalis	0.39 ± 0.24^{b}	0.69 ± 0.17^{b}	5.09 ± 3.01^{a}	0.012
	Gordonibacter_pamelaeae	4.82 ± 5.33	1.63 ± 2.44	4.14 ± 6.29	0.578
K00658(DLST)	Akkermansia_muciniphila	15.03 ± 10.80	53.61 ± 10.63	71.51 ± 70.38	0.102
	Escherichia_coli	9.54 ± 10.18	2.66 ± 3.89	4.36 ± 3.85	0.275
K00382(DLD)	Akkermansia_muciniphila	15.83 ± 12.20^{b}	57.74 ± 18.48^{ab}	77.73 ± 47.57^{a}	0.033
	Enterococcus_faecalis	4.11 ± 6.54	0.87 ± 0.69	13.05 ± 20.24	0.506
	Escherichia_coli	13.17 ± 14.19	5.20 ± 6.24	4.75 ± 3.32	0.299
	Lactobacillus_reuteri	10.63 ± 8.69	56.96 ± 54.61	51.67 ± 21.02	0.101
	unclassified	51.46 ± 28.22	44.38 ± 27.61	68.24 ± 41.91	0.525
K01692(paaF)	Escherichia_coli	7.98 ± 7.00	5.71 ± 7.23	4.90 ± 2.67	0.761
K01782(fadJ)	Escherichia_coli	2.62 ± 2.39	1.98 ± 1.02	0.45 ± 0.11	0.287
K00626(E2.3.1.10)	Escherichia_coli	21.19 ± 21.90	7.32 ± 6.87	4.10 ± 3.39	0.166
	Lactobacillus_crispatus	12.31 ± 6.87	5.23 ± 3.96	9.13 ± 5.10	0.163
	Lactobacillus_helveticus	0.89 ± 2.00	0.32 ± 0.72	0.48 ± 0.71	0.775
	Lactobacillus_johnsonii	32.37 ± 19.52	28.65 ± 18.16	34.94 ± 21.25	0.912
	Lactobacillus_reuteri	17.73 ± 15.48	41.53 ± 63.82	40.18 ± 50.27	0.684
	unclassified	19.49 ± 8.26	29.30 ± 14.02	11.22 ± 5.53	0.066

[a,b] Means within the same row sharing the same superscript letter or bearing no superscript indicate no significant difference ($p > 0.05$), and means with different superscript letters indicate a significant difference ($p < 0.05$). '-' Too low to be calculated. Data are presented as mean ± SD (n = 15). Highlighted numbers indicate *P*-values from *Akkermansia muciniphila*, *Lactobacillus reuteri* and *Enterococcus faecalis*.

[1] Superscript letters indicate the levels in KEGG pathways.

Table S6 Effect of dietary tryptophan levels on the abundance of Trp_low or Trp_high biomarker KOs in different KEGG level 3 pathways.

Pathways[1]	KOs	Species origin	Abundance			P-value
			Control	Trp_low	Trp_high	
**1st Metabolism**						
**2nd Amino acid metabolism**						
**3rd Arginine biosynthesis**	**K01425** (glutaminase)	Akkermansia_muciniphila	16.07 ± 12.02	62.50 ± 23.53	73.70 ± 66.11	0.095
		Lactobacillus_reuteri	15.90 ± 9.32[b]	99.45 ± 94.66[a]	128.56 ± 55.33[a]	0.046
	**K23265** (phosphoribosylformylglycinamidine synthase subunit PurQ)	Akkermansia_muciniphila	19.00 ± 16.37[b]	62.68 ± 14.82[a]	96.38 ± 67.24[a]	0.043
		Enterococcus_faecalis	.046 ± 0.16	0.73 ± 0.46	4.93 ± 4.10	0.104
**2nd Carbohydrate metabolism**						
**3rd Pentose phosphate pathway**	**K01783** (ribulose-phosphate 3-epimerase)	Akkermansia_muciniphila	15.70 ± 12.90	48.48 ± 3.58	72.54 ± 67.49	0.099
**2nd Energy metabolism**						
**3rd Oxidative phosphorylation**	**K00425** (cytochrome bd ubiquinol oxidase subunit I)	Akkermansia_muciniphila	19.22 ± 13.63	57.55 ± 23.70	75.13 ± 63.05	0.198
		Lactobacillus_reuteri	17.39 ± 9.02	63.79 ± 59.62	67.70 ± 24.71	0.269
**2nd Metabolism of cofactors and vitamins**						
**3rd Folate biosynthesis**	**K14652** (3,4-dihydroxy 2-butanone 4-phosphate synthase / GTP cyclohydrolase II)	Akkermansia_muciniphila	17.21 ± 11.07	63.80 ± 11.94	93.71 ± 95.83	0.108
		Lactobacillus_reuteri	26.71 ± 26.54	93.48 ± 110.90	157.24 ± 61.81	0.119
**3rd Nicotinate and nicotinamide metabolism**	**K03472** (D-erythrose 4-phosphate dehydrogenase)	Akkermansia_muciniphila	17.32 ± 10.80	59.83 ± 18.25	74.83 ± 64.65	0.083
		Enterococcus_faecalis	0.47 ± 0.38[b]	1.56 ± 0.85[b]	5.30 ± 3.57[a]	0.046
		Lactobacillus_reuteri	9.46 ± 5.93	62.05 ± 58.63	71.88 ± 34.73	0.060
**1st Cellular Processes**						
**2nd Cell growth and death**						
**3rd Cell cycle Caulobacter**	**K01338** (ATP-dependent Lon protease)	Unclassified	48.40 ± 14.49[a]	60.34 ± 7.03[a]	28.37 ± 8.27[b]	0.003

Table S6 Effect of dietary tryptophan levels on the abundance of Trp_low or Trp_high biomarker KOs in different KEGG level 3 pathways. (Continued)

Pathways	KOs	Species origin	Abundance			P-value
			Control	Trp_low	Trp_high	
**1st Genetic Information Processing**						
**2nd Replication and repair**						
**3rd DNA replication**	**K02316**	Enterococcus_faecalis	0.44 ± 0.28^{b}	0.80 ± 0.31^{b}	4.08 ± 2.35^{a}	0.015
	(DNA primase)	Lactobacillus_reuteri	10.85 ± 9.37	53.84 ± 51.87	58.97 ± 16.90	0.073
**2nd Translation**						
**3rd Aminoacyl tRNA biosynthesis**	**K01867**	Akkermansia_muciniphila	17.77 ± 14.67	51.12 ± 9.35	55.29 ± 58.23	0.251
	(tryptophanyl-tRNA synthetase)	Enterococcus_faecalis	1.43 ± 1.76^{b}	1.84 ± 0.56^{b}	5.99 ± 2.61^{a}	0.018
		Lactobacillus_reuteri	12.60 ± 11.23	85.14 ± 80.01	92.08 ± 31.20	0.055

[a,b] Means within the same row sharing the same superscript letter or bearing no superscript indicate no significant difference ($p > 0.05$), and means with different superscript letters indicate a significant difference ($p < 0.05$).

[1] The numbers ahead of the pathways indicate its hierarchy in KEGG.

'-' Too low to be calculated. Data are presented as mean ± SD (n = 15).

P-values < 0.05 are highlighted.

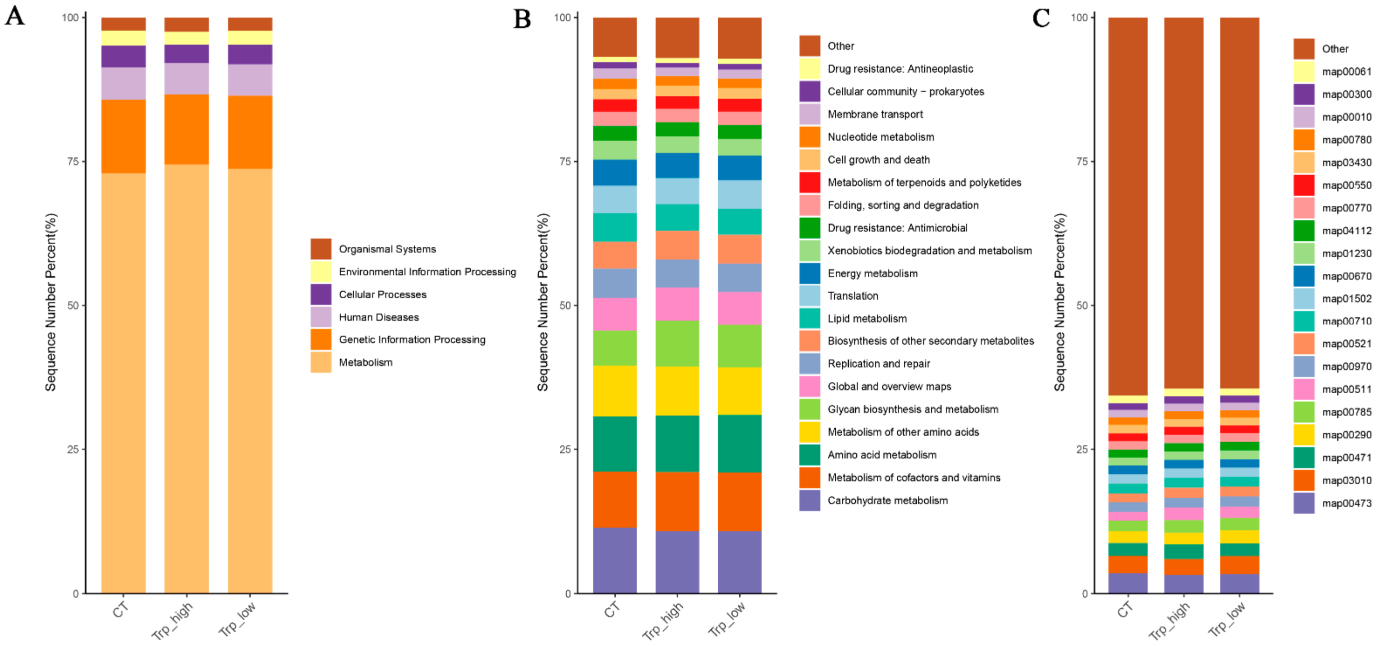

Figure S1 KEGG pathway composition at different levels. (A) Group mean barplot of level 1. (B) Group mean barplot of level 2. (C) Group mean barplot of level 3 (pathway map).

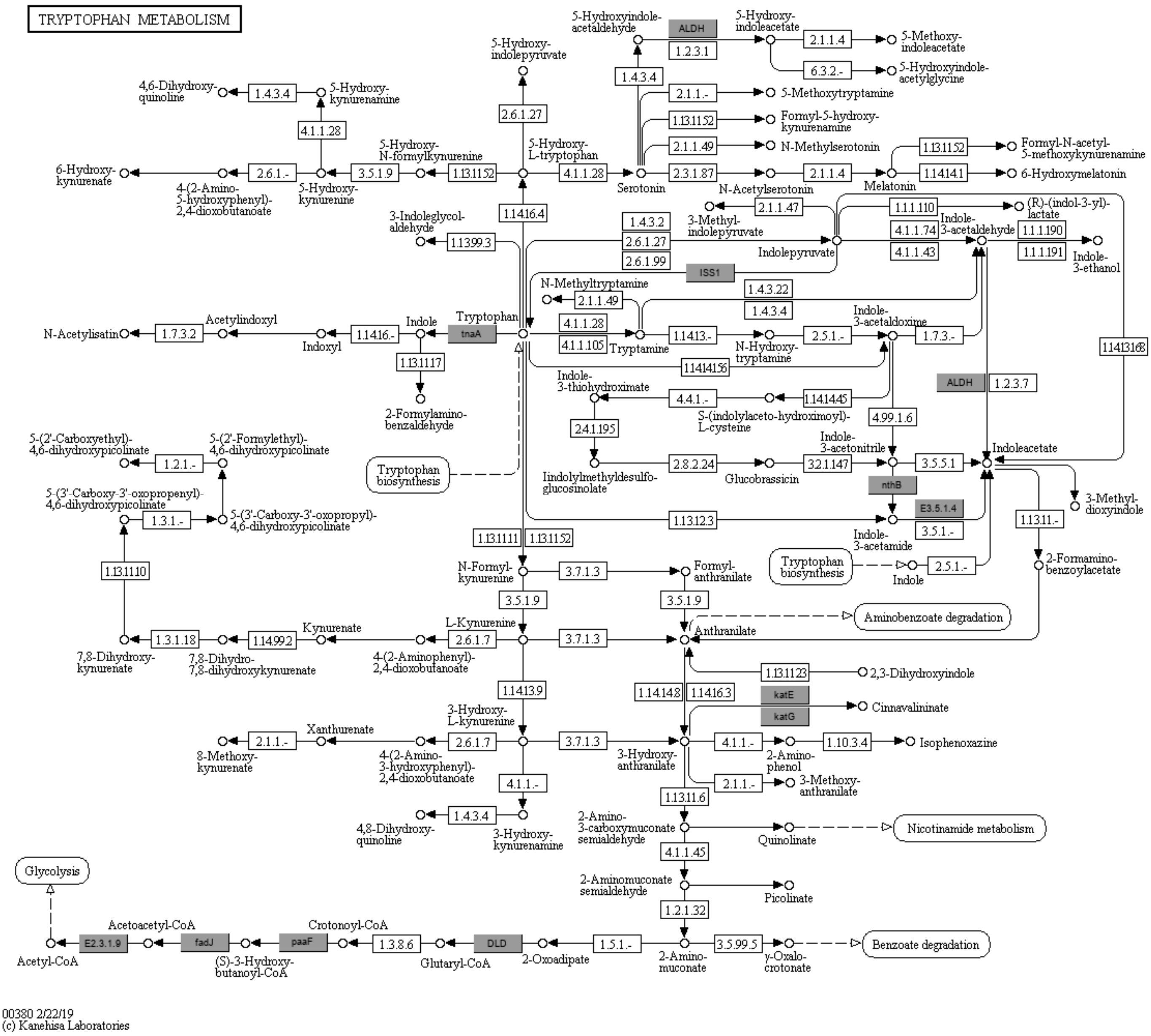

Figure S2 Tryptophan metabolism KEGG pathway map (map00380, level 3) in Amino acid metabolism (level 2) of Metabolism (Level 1). Dark gray boxes represent the KOs detected in the samples of this study and were detailed in Table S5.
